# Successful Childbirth During Satralizumab Treatment in Neuromyelitis Optica Spectrum Disorder

**DOI:** 10.7759/cureus.55010

**Published:** 2024-02-27

**Authors:** Saki Nakashima, Akihito Hao, Naohiro Uchio, Hideyuki Matsumoto

**Affiliations:** 1 Neurology, Mitsui Memorial Hospital, Tokyo, JPN

**Keywords:** pregnancy, perinatal period, parturition, neuromyelitis optica spectrum disorder, satralizumab

## Abstract

A 40-year-old woman with neuromyelitis optica spectrum disorder (NMOSD) and anti-aquaporin 4 antibodies suffered three NMOSD episodes between 35 and 37 years of age. Despite treatment with prednisolone and azathioprine, her condition repeatedly relapsed. We introduced satralizumab, targeting interleukin-6 receptors, which stabilized her condition. At the age of 38, she became pregnant and delivered a healthy baby at 38 weeks. Post delivery, both mother and child stayed healthy with no NMOSD relapses. This case illustrates the efficacy and safety of satralizumab in managing NMOSD, especially for women in their reproductive years who are planning pregnancy.

## Introduction

Neuromyelitis optica spectrum disorder (NMOSD) is an inflammatory central nervous system (CNS) syndrome distinct from multiple sclerosis and is associated with serum anti-aquaporin-4 immunoglobulin G antibody (anti-AQP4-antibody) [1]. The prevalence of NMOSD is higher in women, with a female-to-male ratio of up to 9:1, and the average age of onset is approximately 30-40 years [2-4], indicating that many women could develop NMOSD during their reproductive ages. AQP4 is found in the syncytiotrophoblast layer of the human placenta [5]. The expression of placental AQP4 peaks during mid-gestation and gradually diminishes as pregnancy progresses. Severe inflammation in placentas leads to necrosis, causing fetal death or spontaneous abortion. Pregnancy and the postpartum period are also associated with an increased relapse rate, with the highest annualized relapse rate occurring during the three months after parturition [6,7]. Therefore, stabilizing disease activity before pregnancy is essential for successful childbirth.

The pathogenesis of NMOSD involves an IL-6-dependent B cell subpopulation, offering a therapeutic strategy for targeting IL-6R signaling [8]. Satralizumab is a novel humanized IgG anti-IL-6 receptor monoclonal recycling antibody used in patients with NMOSD who are positive for anti-AQP4 antibody. These anti-AQP4 antibody target astrocytes and cause severe damage, including demyelination, to the optic nerve, spinal cord, and brain. Clinical trials have demonstrated that satralizumab treatment significantly reduces the annualized relapse rate in patients with NMOSD [9,10], and satralizumab has FDA approval for NMOSD. However, the safety of satralizumab in pregnant patients is yet to be established, though another anti-IL-6 receptor monoclonal antibody showed no increased rates of spontaneous abortion or congenital abnormalities with a rheumatic disease [11]. In this report, we present a case of a patient with NMOSD who successfully delivered a baby while undergoing immunosuppressive treatments, including satralizumab.

## Case presentation

**Figure 1:**
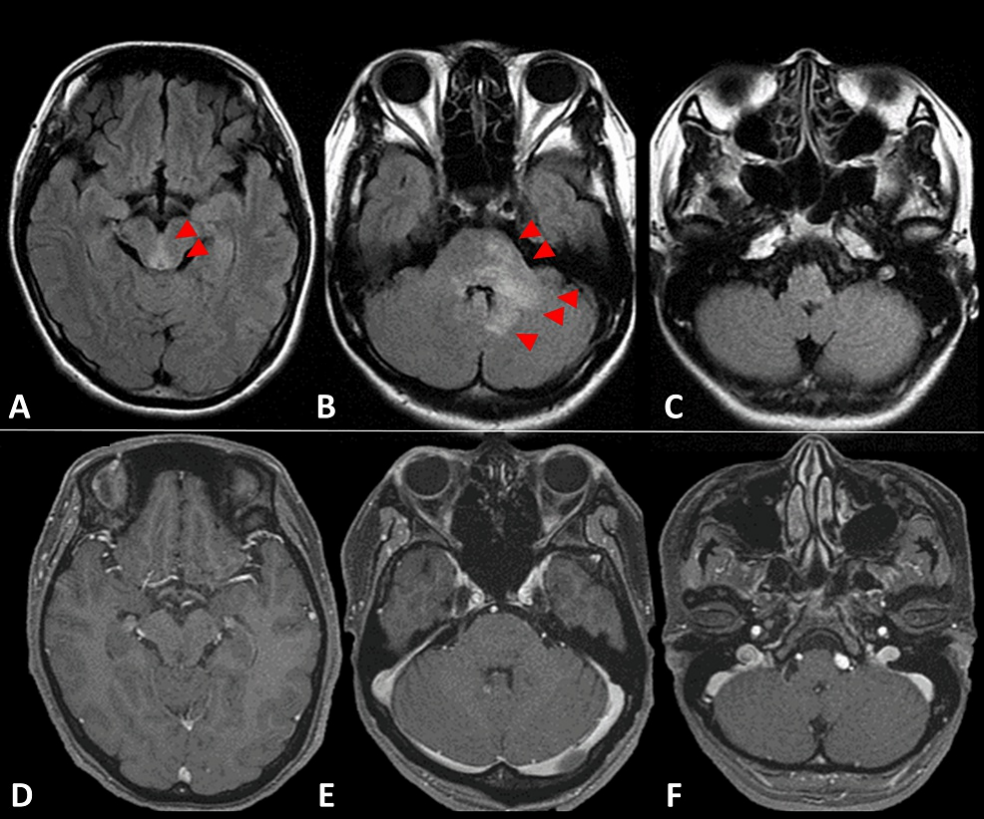
Brain MRI images at first attack of neuromyelitis optica spectrum disorder (NMOSD) MRI FLAIR of the midbrain level (A), pons level (B), and medulla level (C) showing hyperintense lesions extending from the pons to the cerebellum (red arrows); MRI Gd-T1WI (D-F) images showing no any gadolinium enhancement at any level. FLAIR: fluid-attenuated inversion recovery; Gd-T1WI: Gadolinium-enhanced T1-weighted

A 35-year-old woman with no pertinent medical history presented with her inaugural attack of NMOSD, manifesting diplopia and bilateral ptosis, leading to her admission to the neurology department. Brain MRI revealed fluid-attenuated inversion recovery (FLAIR) hyperintense lesions extending from the pons to the cerebellum without gadolinium (Gd) enhancement at the first attack. (Figure 1). Cerebrospinal fluid examination indicated an increased cell count (14 cells/µL; 13 monocytes, one multinucleated cell). A blood examination confirmed the presence of anti-AQP4 antibody. Given the seropositivity for anti-AQP4 antibody, acute brainstem syndrome, and the exclusion of alternative diagnoses, the patient received a diagnosis of NMOSD. Intravenous methylprednisolone therapy (IVMP) was initiated with a dosage of 1000 mg per day, administered over three days each week, for a total of three weeks. Subsequently, her symptoms improved, and she was initially given a prescription for oral prednisolone (PSL) at a daily dose of 20 mg, which was maintained for about three weeks. After this period, the dosage was decreased to 15 mg per day and continued for another month. Following that, while confirming that the disease remained in remission, the dosage was gradually reduced to 8 mg per day.

**Figure 2:**
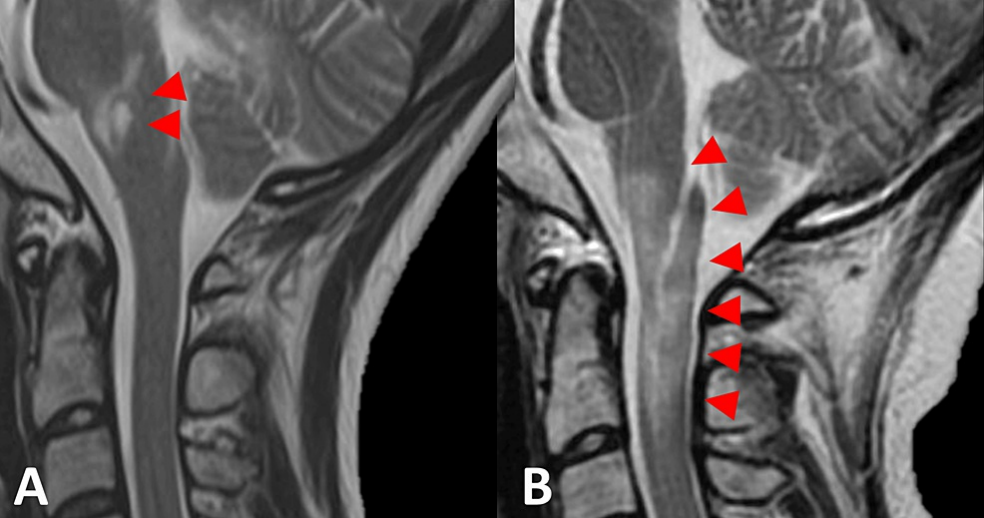
Cervical MRI T2WI at first and second attack Cervical MRI showed T2WI high signal at the level of medulla level (A) at the first attack, and cervical MRI revealed T2WI hyperintense lesions from the medulla to the cervical vertebral bodies 2 level in the spinal cord at the second attack. T2WI: T2-weighted imaging

At the age of 36 years, she reported numbness spreading to both upper limbs and the posterior neck-her second attack. One month later, the patient experienced sensory disturbances in both hands, gait disturbances, and dysuria. Spinal MRI revealed T2-weighted hyperintense lesions from the medulla to the C2 level in the spinal cord (Figure 2), which showed improvement post IVMP therapy. The patient was discharged with a maintenance dose of PSL at 20 mg. To facilitate a safe tapering of PSL, azathioprine (AZA) at 50 mg was introduced.

**Figure 3:**
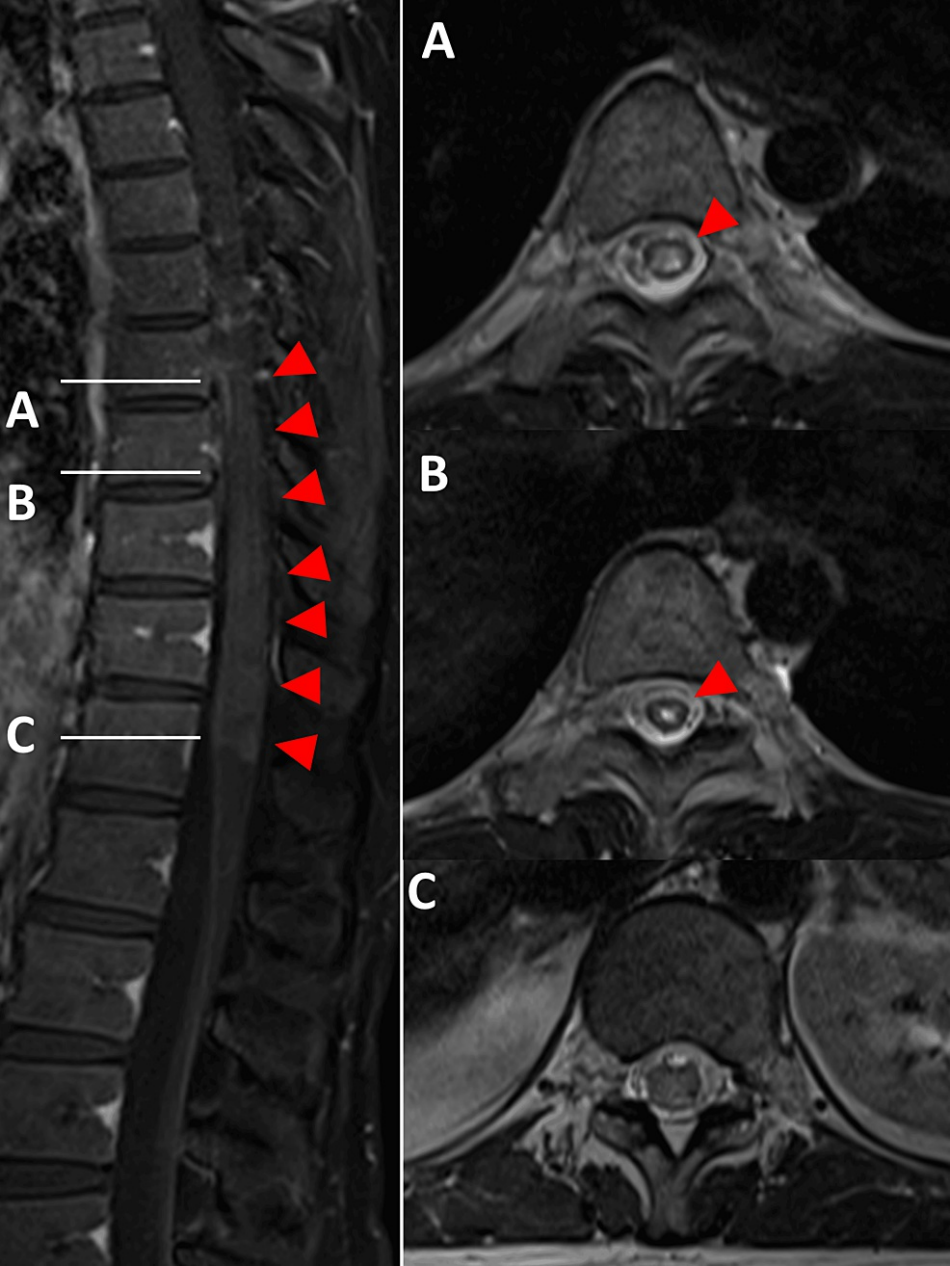
Thoracic MRI Gd-T1WI in the sagittal plane and T2WI in the axial plane at third attack Thoracic MRI revealed longitudinal lesions with gadolinium enhancement (red arrows), extending from the thoracic vertebral bodies (T) 8 to T12 levels. The respective heights indicated in the sagittal section of T2WI correspond to axial slices at the following levels: (A) is at T8, (B) is at T9, and (C) is at T12. Gd-T1WI: Gadolinium-enhanced T1-weighted; T2WI: T2-weighted imaging

At the age of 37 years, however, while gradually tapering PSL to 5 mg, she experienced numbness in the abdomen and bilateral thighs, constituting the third attack. Over the subsequent four-month follow-up period, her ability to walk gradually diminished. Spinal MRI revealed longitudinal lesions with gadolinium enhancement, extending from the T8 to T12 levels (Figure 3).

Despite the severity of the third episode, IVMP therapy significantly ameliorated the symptoms of the patient. Due to recurrent relapses following treatment with PSL and AZA, we administered satralizumab as a subcutaneous injection of 120 mg. Informed consent was obtained from the patient and her husband, following a comprehensive explanation of the potential risks of satralizumab, including maternal and fetal death due to infection.

Six months after her last relapse, she received our approval to proceed with pregnancy. One month later, while on treatment with PSL (5 mg), AZA (100 mg), and satralizumab, she had a successful pregnancy and delivered a healthy girl via normal vaginal parturition at 38 weeks of gestation. The patient was still on a regimen that included PSL, AZA, and satralizumab for her NMOSD treatment. While PSL and AZA are not particularly problematic regarding transfer into breast milk, the evidence for satralizumab's safety in lactation is not yet established. Due to concerns about the potential for postpartum infections, the patient chose not to breastfeed her child and opted for formula milk. No relapses occurred, and her child has grown without any issues to date, i.e., one year after childbirth. 

**Figure 4:**
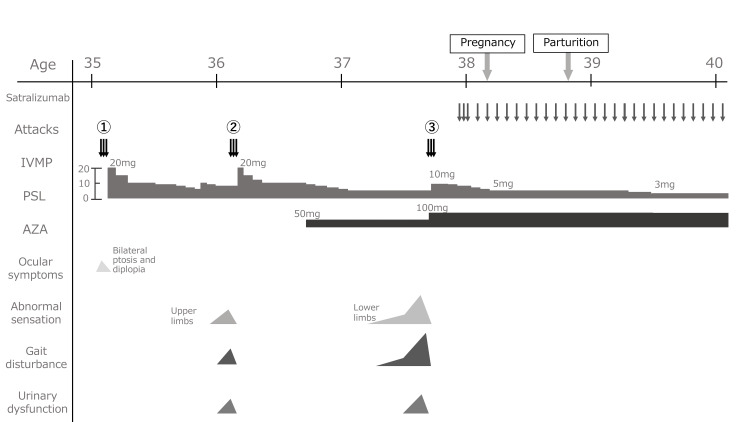
Clinical course following the onset of NMOSD The first episode of NMOSD was treated with intravenous methylprednisolone (IVMP) therapy, followed by prednisolone (PSL) owing to the desire for pregnancy. Azathioprine (AZA) was initiated after the second attack, and satralizumab was administered after the third attack. After achieving disease stability (i.e., after six months after the start of satralizumab), she was given the go-ahead for pregnancy under treatment with PSL 5 mg, AZA 100 mg, and satralizumab. One month after obtaining permission, pregnancy was observed. No relapses occurred in the perinatal period. At 38 weeks’ gestation, the patient delivered a healthy baby. Three months after parturition, during the period of high risk of relapse, PSL was gradually tapered to 3 mg. She and her child have had no problems to date, one year after parturition. NMOSD: neuromyelitis optica spectrum disorder

The clinical course of the patient is depicted in Figure 4.

## Discussion

The patient encountered three NMOSD attacks under PSL and AZA between 35 and 37 years of age, prompting satralizumab intervention. At age 38, six months post stabilization, pregnancy ensued, leading to conception the subsequent month and a healthy delivery at 38 gestational weeks. Post-partum, no relapse occurred, and the child thrived without complications

The ultimate NMOSD attack transpired at a PSL dosage of 5 mg. While PSL serves as a common NMOSD maintenance treatment in select regions, its prolonged use links to severe complications, including infection, moon face, weight gain, gastric ulcers, hypertension, hyperglycemia, cataracts, glaucoma, osteoporosis, and psychosis [12,13]. Significantly, a Japanese study illustrated that PSL doses could be tapered in satralizumab-treated NMOSD patients without escalating annualized relapse rates [14]. Indeed, in our patient, PSL dosage decreased to 5 mg during pregnancy and further dwindled to 3 mg post-partum.

The first reported case of childbirth in a patient with NMOSD treated with satralizumab demonstrated that maternal serum concentration of satralizumab was 9.59 μg/mL on the day of parturition. In contrast, satralizumab did not significantly transfer to the umbilical cord blood or child, with serum concentrations undetectable on the day of parturition (< 0.200 μg/mL) [15]. The transfer rate of satralizumab from mother to child is estimated to be less than 2%. Our case represents the second reported instance. However, unlike the first case [15], our treatment approach did not include the use of IVIg; we only used a low dose of steroids and AZA alongside satralizumab. We believe this highlights the efficacy of the treatment more strongly.

Similar to satralizumab, a earlier case report investigated the safety of tocilizumab, another IL-6 receptor inhibitor, during the perinatal period [16]. The transfer rate of tocilizumab from mother to child is estimated to be approximately 14% on the day of parturition. As there were no issues with the growth and development of the child during tocilizumab treatment, the authors concluded that treatment with tocilizumab could be managed during the perinatal period. Comparing the reports by Yoshida et al. [15] and Saito et al. [16], satralizumab appears to be safer than tocilizumab regarding infection risk in children. The extremely low transfer of satralizumab to the child might be explained by the lower placental transfer of subclass IgG2 in satralizumab compared to that of subclass IgG1 in tocilizumab.

In the first reported case, Yoshida et al. also stated that while the concentrations in maternal serum ranged from 4.69 to 10.3 μg/mL, the concentrations in breast milk were below detection sensitivity (< 0.200 μg/mL) [15]. The transfer rate of satralizumab into breast milk was estimated to be < 2%. When administered orally, the small dose of satralizumab in breast milk is assumed to be digested and hardly absorbed. Although the patient in the current report avoided breastfeeding, the extremely low transfer rate supports the safety of breastfeeding during satralizumab use.

In the case reported by Yoshida et al., however, the patient received monthly IVIg at a dosage of 400 mg/kg/day for five successive days per month, starting from the onset of the disease until breastfeeding graduation, including the perinatal period [15]. This regimen has led to more than 20 IVIg treatments. IVIg therapy may have been effective in improving symptoms and preventing relapse and may also have played an important role in preventing infectious diseases. Therefore, owing to uncommon therapies, the risk of infection is lowered. In contrast, the patient in the current study used only immunosuppressive medications such as PSL, AZA, and satralizumab. Therefore, our patient is the first case of successful childbirth under purely immunosuppressive conditions using satralizumab. Our patient had no relapse, and the child too had no problems to date, that is until one year old. However, further research is needed to investigate the safety of satralizumab during the perinatal period, which includes a maternal relapse, maternal infection, fetal death, spontaneous abortion, congenital abnormalities, infection, breastfeeding, and child growth and development.

## Conclusions

Herein, we reported the case of a 38-year-old woman with NMOSD who successfully became pregnant and gave birth after treatment with PSL (5 mg), AZA (100 mg), and satralizumab. Based on the present case and the existing literature, it is suggested that satralizumab has the potential to effectively stabilize disease activity before pregnancy and could be considered a safe option for patients with NMOSD who are planning for childbirth. However, further accumulation of similar cases is required to establish the safety of satralizumab during the perinatal period.
